# 

**Published:** 1896-08-15

**Authors:** 


**Notices of Births, Deaths, and Marriages** are inserted in this column at a charge of 2s. 6d. for an announcement not exceeding four lines.

### NOTICE TO CORRESPONDENTS.

All MS., Letters, Books for Review, and other matters intended for the Editor should be addressed THE EDITOR, THE LODGE, PORCHESTER SQUARE, LONDON, W.

The Editor cannot undertake to return rejected MS., even when accompanied by stamped directed envelope.

**Notice to Advertisers and Subscribers.**

All Advertisements, Orders for Copies of THE HOSPITAL and Business Communications should be addressed to **THE MANAGER, (Not to The Editor).** THE HOSPITAL, 428, Strand, London, W.C.

The Cost of Subscription, post free, is as follows:—Per week, 2½d.; per quarter, 2s. 8d.; per half-year, 5s. 3d.; per annum, 10s. 6d. Foreign:—Per Annum, 15s. 2d.; payable, in all cases, in advance.

Price 5s.

**"Burdett's Hospitals and Charities, 1896."**

Seventh year of publication. Over 1,000 pp. Scarlet cloth, gilt lettered. Price 5s. A most complete guide to British, Colonial, Indian, and American Hospitals, Dispensaries, Nursing and Convalescent Institutions, and Asylums. A few complete sets of the preceding six issues of the "Annual" may still be obtained on application to the Publishers, THE SCIENTIFIC PRESS (Limited), 428, Strand, London, W.C.

**NOTICE.—Vol XIX. of "The Hospital" (October, 1895, to April, 1896), in handsome cloth binding, is on sale at the Office of this Journal. Price 7s. 6d. Cases for binding the Numbers contained in this Volume. 1s. 6d. Index to Vol. XIX., 2½d., post free. The Monthly Part for August is now ready. Price 6d., by post 8d.**

### SCRAPS AND GLEANINGS.

A new operating theatre for the City of Dublin Hospital is under consideration.

A new infirmary is shortly to be erected by the Wakefield Board of Guardians.

The New Hospital Fund for rebuilding the Bedford General Infirmary now amounts to £22,559.

The collections being made towards founding a cottage hospital at Moffat, N.B., have reached the sum of £1,250.

A donation of £500 has been sent to the managers of the Samaritan Hospital, Belfast, towards the erection of a new wing.

Dr. Samuel Wilks, F.R.S., has become a vice-president of the Bethnal Green Free Library, in place of the late Sir Russell Reynolds.

The Sydney (N.S.W.) Hospital Saturday Fund Association have collected this year £3,965, against £3,514 in 1894-5, and £1,919 in 1893-4.

Another decrease in street collections. The receipts from this source on Hospital Saturday at Hull have been as follows: 1894, £530; 1895, £496; 1896, £427.

Her Majesty the Queen has graciously promised to contribute £50 towards the extension and improvement fund of Queen Charlotte's Lying-in Hospital.

The first Hospital Saturday held in Aberdeen has resulted in the distribution of £950 amongst the various hospitals and kindred institutions of that town.

The opening of the new wards at the Walsall and District Cottage Hospital, erected to replace those demolished during the gale in March, 1895, took place recently.

The payments by patients with tickets of recommendation at the Hull and East Riding Convalescent Home have been reduced from 5s. a week for adults and 2s. 6d. for children, to 4s. and 2s. respectively.

It was necessary during the past year to draw upon the small reserve fund of the Uckfield Cottage Hospital to the extent of £25 in order to meet the expenditure. Thirty-seven cases were admitted during the year.

The Oldbury Hospital Saturday Committee have collected £547 odd this year. As the expenses are defrayed by special donations, the whole of this sum is handed over to the institution on whose behalf the money is collected.

Owing to the alterations made at the Walsall and District Hospital, by which 16 beds have been added to the 50 formerly provided, the hospital authorities state that an additional income of £1,000 a year will be required.

The result of the house-to-house and street collections made at Bury St. Edmunds in aid of the Suffolk General Hospital is that a sum of £84 has been added to the funds of that institute. This is about £2 10s. less than last year.

A novel result of treatment received at a hospital is a bazaar promoted recently by a private patient at the Hull Orthopædic and Women's Hospital, as a means of showing her gratitude to and admiration for that institution.

The annual report of the Weymouth and Dorset County Royal Infirmary shows that 191 in and 823 out-patients were treated during the year ended July, 1896. The income (including £675 legacies) amounted to £1,224, and the expenditure to £572, £648 being invested.

Towards a deficit of £800 on the accounts of the East Suffolk and Ipswich Hospital for the year 1895, Captain Pretyman, M.P., has sent a cheque for £100. At the same time he suggests the formation of a working men's committee to make workshop and other collections.

The finances of the Association for the Oral Instruction of the Deaf and Dumb, as shown by the latest balance-sheet, that for 1895, are not in a flourishing condition. The income amounted to £1,192 and the expenditure to £2,058, increasing the overdraft at the bankers to £2,470.

At the Coombe Lying-in Hospital, Dublin, 462 patients were admitted last year to the labour wards, 256 to the chronic wards, 1,513 maternity cases were attended at their own homes, 2,083 attended at the special dispensary for diseases of women and children, and 8,883 at the general dispensary.

The Woking, Horsell, and Woodham Cottage Hospital expended during the twelve months ended April 30th, 1896, £359, and received on general account £492, and on building fund account £56. 53 in and 171 out patients were treated, as compared with 24 and 50 respectively during the year 1894 5.

The Red Cross Germicide Company has obtained the gold medal and diploma for their "Perfume Germicide" at the International Life-Saving Exhibition, London; these being the only awards offered for disinfectants. They have received the same distinction for their "Dr. Siemon's Pocket Inhaler."

To Caffyn's Liquor Carnis was decreed the only medal for specific merit at the Chicago Exhibition, 1893, in competition with the world. They were also awarded a medal and diploma for their preparations of Bone-marrow, Virol, Marrol, and Virol sans Sucre, "for excellence in usefulness and also for nutritive value."

The committee of the Yarmouth Hospital state that, owing to the opening of the new female ward, an additional income of £300 has been necessitated. During the year ended May 31st, 1896, 318 in and 4,196 out patients were treated. The expenditure during the same period, viz., £2,022, exceeded the income by £10.

Two thousand three hundred and eighty-nine patients were treated at the Liverpool Convalescent Institution at Woolton during 1895. Of this number 505 were nominated by subscribers and donors to free beds; 460 were received through the medium of subscribers' "recommendation" forms, each paying 5s. per week; while the remaining 1,420 were received from local hospitals and private homes.

An excellent method of driving home to the minds of the inhabitants of the district surrounding a hospital the fact that they do not contribute as they should to its funds is that used in the special appeal recently issued by the Newcastle Royal Infirmary. A statement has been made out showing for each adjacent district the number of patients sent to the hospital, the estimated cost of those patients, the amount of contributions received, and the profit or loss (generally loss) on the transaction.

### The Student of Medicine.

### APPOINTMENTS.

J. Harold Bailey, M.B., Ch.B.Vict., appointed Lecturer in Physiology and Hygiene at the Salford Royal Technical Institute. Mr. J. C. Baker, appointed Medical Officer for the Workhouse of the Hambledon Union. Aslett Baldwin, F.R.C.S.Eng., L.R.C.P.Lond., appointed Casualty Surgical Officer to the Middlesex Hospital, W. G. Batters, M.B., M.S.Edin., appointed Medical Officer of Health to the Kempston Urban District Council. H. A. Caley, M.D.Lond., M.R.C.P., appointed Physician in charge of Out-patients to St. Mary's Hospital. A. Chaplin, M.D.Cantab., M.R.C.P., appointed Physician to the Metropolitan Dispensary, Cripplegate. F. W. S. Culhane, M.R.C.S.Eng., L.S.A., appointed Medical Officer for the Workhouse of the Hastings Union. F. J. Falvey, L.R.C.P., L.R.C.S.Edin., appointed Physician to the Kerry Fever Hospital. Mr. J. D. Hessey, appointed Medical Officer for the First District of the Hastings Union. Dr. Holmes, appointed Medical Officer for the Parishes of Breadsall and Little Eaton of the Shardlow Union. F. A. Juckes, M.B., C.M.Edin., appointed Medical Officer for the Workhouse and the First of the Horsham Union. Mr. T. McCormack, appointed Dispensary Medical Officer for Spiddal. J. P. McLaren, M.B., appointed Medical Officer for the Claygate District of the Kingston Union. A. W. C. Peskett, M.A., M.B., B.C.Cantab., appointed Honorary Medical Officer to the Hospital for Sick Children, Nottingham. K. Roberts, M.R.C.S.Eng., L.R.C.P.Lond., appointed Medical Officer for the Shillington District of the Ampthill Union. J. E. Sharpley, M.R.C.S., L.R.C.P.Lond., appointed Medical Officer for the Kirton-in-Lindsey District of the Blyboro' Union. W. N. Thursfield, M.D.Edin., M.R.C.S.Eng., D.P.H.Camb., appointed Medical Officer of Health to the Dawley Urban District Council. A. Trethewy, M.B., B.A.Camb., M.R.C.S.Eng., F.R.C.P.Lond., appointed House Surgeon to the Warneford Hospital, Leamington. I. B. Yeo, M.D., F.R.C.P.Lond., appointed Professor of the Principles and Practice of Medicine at King's College, London.

### PASS LISTS.

### Conjoint Board in Scotland.

At the July sittings of the Scottish Conjoint Examining Board, held in Glasgow, the following candidates passed the respective examinations, those marked with an asterisk (*) passing with distinction:—

First Examination, Five Years' Course.—D. S. E. Macnab, J. Noble, D. Crombie, P. H. Rainbird, T. M'Donagh, A. E. Ward, J. B. O'Mahony, T. B. Adam, J. B. Walker, J. T. Griffiths, Mary E. Potter, G. Evans, T. Little, R. E. Naish, H. M. Roberts, A. S. Sieger, W. J. Buckmaster, J. E. M'Intyre, S. J. Farries, *A. Scoular, Alice Miles, T. Henderson, D. Fitzgibbon, J. F. Shone, L. M'Sherry, A. M'Innes, R. E. Moorhead, F. A. Gray, A. F. Fleming, G. J. Campbell.

First Examination, Four Years' Course.—Louise Fox, A. N. Davies, A. Dougall, T. E. Saxby, H. V. Craster, T. W. Colthurst, G. G. Wilson, D. K. Edwards.

Second Examination, Five Years' Course.—W. M'Farlane, H. A. Marquis, Jeannie H. Traill, S. J. Smith, T. K. Thomson, R. Stewart, A. J. Wilson, A. F. Jack.

Second Examination, Four Years' Course.—J. Davies, J. Elliott, G. L. Jones, E. A. Kirkwood, G. R. Hasland, *H. O. Jones, *F. G. Henderson, E. A. Clark, A. L. Brown, J. Farmer, T. H. O'Reilly, T. S. Ross, D. C. Rowlands, W. Beck, A. W. Swettenham, H. L. Phelps, A. N. Davies, D. S. C. Reid.

Third Examination, Five Years' Course.—S. V. Robinson, H. Chaffer, E. H. Sheldon, J. R. Riddell, V. Bateson.

Final Examination.—J. Battersby (with honours), W. G. Pritchard, H. M'Kay, R. A. Cameron, W. H. Brooks, R. P. Graham, A. G. Johnson, K. T. Matthew, J. H. Powell, J. H. Hall, J. E. Harburn, M. M'Sherry, G. M. Speers, J. Robertson, C. W. Davidson, J. C. Mackenzie, C. A. K. Renshaw, M.A., J. L. Cormac, Mary S. S. Coghill, C. Retallack, A. S. Marr, J. H. Wigham, N. C. Boyle.

### BOOKS RECEIVED.

BAILLIERE, TINDALL, AND COX.

"The Imperial Health Manual." Edited by Anthony Roche, M.R.C.S.I.

WM. HEINEMANN.

"The Biological Problem of To-day." By Professor Dr. Oscar Hertwig. Authorised Translation by P. Chalmers Mitchell, M.A.

JOHN WRIGHT AND CO.

"Angio-Neurosis." By W. Ramsay-Smith, M.B., C.M., B.Sc.

"The Surgery of the Chest." By Stephen Paget, F.R.C.S.

J. AND A. CHURCHILL.

"A Short Manual for Monthly Nurses." By Charles J. Cullingworth, M.D., F.R.C.P. Revised by M. A. Atkinson.
"Surgical Emergencies." By Paul Swain, F.R.C.S.

JOHN BALE AND SONS AND HENRY KIMPTON, LONDON.

"The Causes and Treatment of Rheumatoid Arthretis." By Samuel Hyde, M.D.

SAMPSON LOW, MARSTON, AND CO., LONDON.

"Twentieth Century Practise and International Encyclopedia of Modern Medical Science." By leading authorities of Europe and America. Edited by T. Steadman, M.D.

MACMILLAN AND CO., LONDON.

"Deformities, a Treatise on Orthopædic Surgery intended for Practitioners and Advanced Students." By A. H. Tubbey, F.R.C.S.

**Periodicals and Pamphlets.**—Columbus Medical Journal, Intercolonial Medical Journal of Australasia, Clinical Journal, Woman's Signal, Pediatrics, Australasian Medical Gazette, Medical Week, Edinburgh Medical Missionary Society Quarterly Paper, Guy's Hospital Gazette, St. Bartholomew's Hospital Journal, Zenana, Medical Press, Invention, Sun, New York Medical Journal, International Medical Magazine, Literary Digest, Medical Pioneer, Hermes, Electrical Review, The Planter, London, Leisure Hour, Sunday at Home, Girl's Own Paper, Boy's Own Paper, Nursing Record, Scalpel, Therapist, English Illustrated Magazine, Transactions of the Autumn Congress of the Vegetarian Federal Union, Charlotte Medical Journal, Industries and Iron, Monthly Homœopathic Review, Tri-State Medical Journal, Charity Organisation Review, Bristol Medico-Chirurgical Journal, The Westminster Review.

